# Grafting: A Technique to Modify Ion Accumulation in Horticultural Crops

**DOI:** 10.3389/fpls.2016.01457

**Published:** 2016-10-21

**Authors:** Muhammad A. Nawaz, Muhammad Imtiaz, Qiusheng Kong, Fei Cheng, Waqar Ahmed, Yuan Huang, Zhilong Bie

**Affiliations:** ^1^College of Horticulture and Forestry Sciences, Huazhong Agricultural University/Key Laboratory of Horticultural Plant Biology, Ministry of EducationWuhan, China; ^2^Department of Horticulture, University College of Agriculture, University of SargodhaSargodha, Pakistan; ^3^Microelement Research Center, College of Resources and Environment, Huazhong Agricultural UniversityWuhan, China; ^4^United States Agency for International Development (USDA) and Cultivating New Frontiers in Agriculture (CNFA)Lahore, Pakistan

**Keywords:** grafting, rootstock, scion, root growth, ion uptake, miRNAs, utilization efficiency, transgrafting

## Abstract

Grafting is a centuries-old technique used in plants to obtain economic benefits. Grafting increases nutrient uptake and utilization efficiency in a number of plant species, including fruits, vegetables, and ornamentals. Selected rootstocks of the same species or close relatives are utilized in grafting. Rootstocks absorb more water and ions than self-rooted plants and transport these water and ions to the aboveground scion. Ion uptake is regulated by a complex communication mechanism between the scion and rootstock. Sugars, hormones, and miRNAs function as long-distance signaling molecules and regulate ion uptake and ion homeostasis by affecting the activity of ion transporters. This review summarizes available information on the effect of rootstock on nutrient uptake and utilization and the mechanisms involved. Information on specific nutrient-efficient rootstocks for different crops of commercial importance is also provided. Several other important approaches, such as interstocking (during double grafting), inarching, use of plant-growth-promoting rhizobacteria, use of arbuscular mycorrhizal fungi, use of plant growth substances (e.g., auxin and melatonin), and use of genetically engineered rootstocks and scions (transgrafting), are highlighted; these approaches can be combined with grafting to enhance nutrient uptake and utilization in commercially important plant species. Whether the rootstock and scion affect each other's soil microbiota and their effect on the nutrient absorption of rootstocks remain largely unknown. Similarly, the physiological and molecular bases of grafting, crease formation, and incompatibility are not fully identified and require investigation. Grafting in horticultural crops can help reveal the basic biology of grafting, the reasons for incompatibility, sensing, and signaling of nutrients, ion uptake and transport, and the mechanism of heavy metal accumulation and restriction in rootstocks. Ion transporter and miRNA-regulated nutrient studies have focused on model and non-grafted plants, and information on grafted plants is limited. Such information will improve the development of nutrient-efficient rootstocks.

## Introduction

Grafting is a special type of plant propagation, in which a part of a plant (scion) is joined to another plant (rootstock) for the two parts to grow together and form a new plant. Grafting commonly occurs in nature, and the occurrence of natural grafts might have inspired humans to use grafting in the agriculture sector a thousand years ago (Mudge et al., 2009). The rootstocks used for a specific crop are the crop's close relatives or wild selections (mostly within the *genera*), but natural grafts between different families have also been observed (Warschefsky et al., 2016). Grafting has been practiced in fruit trees for a long time; however, its application in vegetables is relatively new. Various fruit grafting references that appear in the Bibble and ancient Greek and Chinese literature suggest that grafting was utilized in Asia, Europe, and the Middle East in fifth century BC (Melnyk and Meyerowitz, 2015). Similarly, a Chinese book written in first century BC and a Korean book written in seventeenth century AD indicate that grafting was utilized in these periods to produce large gourd fruits (Lee and Oda, 2003). Grafting enhances plant vigor, extends the harvesting period (Lee et al., 2010), improves yield and fruit quality (Huang et al., 2009; Rouphael et al., 2010; Tsaballa et al., 2013), prolongs postharvest life (Zhao et al., 2011), tolerates low and high temperatures (López-Marín et al., 2013; Li et al., 2016b), deals with salinity and heavy metal stress (Santa-Cruz et al., 2002; Estañ et al., 2005; Albacete et al., 2009; Schwarz et al., 2010; Huang et al., 2013a; Wahb-Allah, 2014; Penella et al., 2015, 2016), increases drought and flooding stress endurance (Schwarz et al., 2010; Bhatt et al., 2015), improves water use efficiency (Cantero-Navarro et al., 2016), manages soil-borne and foliar pathogens (Louws et al., 2010; Arwiyanto et al., 2015; Miles et al., 2015; Suchoff et al., 2015), manages root knot nematodes (Lee et al., 2010), controls weeds (Dor et al., 2010; Louws et al., 2010), and produces new plant species (Fuentes et al., 2014). Commercial grafting is currently practiced in a number of plant species, including fruits (citrus, apple, mango, grape, peach, plum, apricot, almond, and cherry), vegetables (watermelon, melon, cucumber, tomato, pepper, eggplant, and bitter gourd), and ornamentals (rose, chrysanthemum, bougainvillea, and bonsai), to obtain economic benefits.

In addition to these commercially important benefits, grafting increases nutrient uptake and utilization efficiency in a number of plant species (Pulgar et al., 2000; Zen et al., 2004; Ahmed et al., 2006, 2007; Albacete et al., 2009, 2015a,b; Lee et al., 2010; Schwarz et al., 2013; Huang et al., 2013b; Esmaeili et al., 2015). The world's nutrient resources are finite (Venema et al., 2011) and thus require justified utilization; moreover, inorganic fertilizers are expensive. Scientists are working to modify the root architecture of cereal crops (monocots) to enhance nutrient uptake and utilization efficiency (Meister et al., 2014; Rogers and Benfey, 2015; Wissuwa et al., 2016). Rootstock grafting is used as an alternative for horticultural crops; appropriate and compatible rootstocks are utilized to improve water and nutrient acquisition and nutrient utilization efficiency (Gregory et al., 2013; Albacete et al., 2015a,b). Nutrient efficiency is a general term that refers to the capacity of a plant to acquire and use nutrients. It is measured by plant dry weight produced per unit of nutrient supplied (g DW/g nutrient supplied). This parameter includes nutrient uptake efficiency and nutrient use efficiency (Gerendás et al., 2008). Although other efficiency indicators, such as nutrient harvest index, nutrient influx rate, and nutrient partitioning, have been proposed (Rengel and Damon, 2008), measurement of plant growth and harvestable yield per unit input of applied fertilizer remains reliable (Venema et al., 2011; Santa-Maria et al., 2015).

Several reviews have been conducted on grafting history, grafting methods, breeding and selection of rootstocks, biotic and abiotic stresses, pathogens, amelioration of heavy metals, and hormonal signaling (Lee, 1994; Lee and Oda, 2003; Davis et al., 2008; Kubota and McClure, 2008; Aloni et al., 2010; Lee et al., 2010; Louws et al., 2010; Pérez-Alfocea et al., 2010; Rouphael et al., 2010; Savvas et al., 2010; Schwarz et al., 2013; Edelstein et al., 2016), but none of these reviews sufficiently elucidated the role of grafting in modifying ion uptake and accumulation. Therefore, the current review summarizes available information on the effects of rootstocks on enhancing nutrient uptake, accumulation, and utilization as well as the mechanism involved. Additionally, other important approaches are presented; these approaches can be combined with grafting to further enhance nutrient acquisition and utilization efficiency.

## Fruit crops

The use of rootstocks in fruit plants affects tree vigor and size, precocity, fruit quality and taste, harvestable yield, resistance to pests, and tolerance against edaphic and environmental conditions by invigorating the scions and increasing nutrient uptake, transport, and utilization efficiency (Albrigo, 1977; Ahmed et al., 2006, 2007; Castle, 2010; Habran et al., 2016). In a previous study, the grafting of kinnow (*Citrus reticulata* Blanco) onto nine different rootstocks increased the concentration of nitrogen (4–18%), phosphorous (11–77%), and potassium (3–43%) in the leaves of the grafted plants; the number of fruits per plant increased to 0.12–5.63 times that of poorly performing rootstock (Ahmed et al., 2007). In another study, total nitrogen accumulation and utilization efficiency differed in various citrus rootstocks; rough lemon (*Citrus jambhiri* Lush.) accumulated a total nitrogen value of 22.1 mg/g DW, whereas Cleopatra mandarin (*Citrus reshni* hort. ex Tanaka) accumulated only 6.1 mg/g DW (Sorgona et al., 2006). Boron concentration in the leaves, stems, and roots of different citrus rootstocks is significantly affected in normal and boron-deficient conditions (Mei et al., 2011). Citrange rootstock [*C. sinensis* (L.) Osb. × *P. trifoliata* (L.) Raf.] is superior to trifoliate orange [*Poncirus trifoliata* (L.) Raf.] under both low and high levels of boron supply. Although the level of boron in different plant parts grafted onto citrange is lower than that grafted onto trifoliate orange, the ratio of semi-bound boron to free boron is much higher in citrange grafted plants; this higher ratio increases boron use efficiency (Liu et al., 2011, 2013). Evidently, uptake capacity is not the only important factor; the transport and reallocation of cellular boron to cell walls also affect rootstock responses to nutrient deficiency. Wang et al. (2016a) recently applied inarching (a special type of grafting) of Carrizo citrange [*C. sinensis* (L.) Osb. × *P. trifoliate* (L.) Raf.] to enhance boron uptake in Newhall navel orange [*C. sinensis* (L.) Osb. cv. Newhall] budded onto trifoliate orange [*P. trifoliata* (L.) Raf.]. Inarching significantly increased the levels of boron in the new leaves, new twigs, scion stem, and new rootstock stem under boron-adequate and boron-deficient conditions. This technique can improve nutrient supply in already growing grafted or self-rooted fruit plants under field conditions.

In apples, Cong et al. (2014) found that rootstocks have different potassium uptakes and accumulation efficiencies; these differences become increasingly pronounced under potassium-deficient conditions. Zarrouk et al. (2005) discovered that the concentrations of macro and micronutrients (N, P, K, Ca, Mg, Fe, Mn, Zn, Na, and Cu) in the leaves and flowers of peach are significantly affected by different rootstocks. Similarly, Amiri et al. (2014) reported that apple rootstocks (M9, MM106, and MM111) increase the concentration of N (5–34%), Mg (5–68%), Zn (10–39%), Fe (4–34%), and Mn (11–70%) and reduce the uptake of K (6–19%), and Ca (10–28%). Trees grafted onto M9 are efficient in N, Mn, and Fe uptake but perform poorly in K and Ca uptake. MM106 rootstock possesses the highest efficiency in P uptake and transport. In this study, the fruit yield of plants grafted onto MM111 increased by 44% compared with plants grafted onto M9 rootstock. Mestre et al. (2015) observed that the concentrations of all macro and micronutrients differ significantly in the leaves of Big Top nectarine (*Prunus persica* var. *nectarina*) grafted onto 12 *Prunus* rootstocks. The rootstocks demonstrate a selective behavior for different elements, such as K, Ca, and Cu.

Rootstocks significantly alter the concentration of nutrients in sweet cherry leaves. The GiSelA 6 rootstock (*Prunus cerasus* × *Prunus canescens*, Gi 148/1) performs well in N, P, K, Zn, B, and Mn uptake and transport, but trees grafted onto it tend to develop Ca, Mg, and Cu deficiencies. Similarly, the *Prunus mahaleb* rootstock performs efficiently in N, P, K, Ca, Mg, Fe, and Cu uptake and transport but performs poorly in Zn, B, and Mn absorption and translocation in plants. The leaves of plants grafted onto *Prunus fruticosa* Prob. show deficiencies in several nutrients (N, P, Ca, Mg, and Cu) and are thus unfit for use (Hrotko et al., 2014). Slowik et al. (1979) studied the avocado (*Persea americana* Mill.) cultivar Hass by using Duke and Topa Topa (avocado rootstocks of Mexican origin) and concluded that only the concentrations of N, P, Mg, Cu, Mn, and Fe are affected by the rootstocks; no significant differences were observed for K, Ca, Na, Cl, and Zn. Sherafati et al. (2011) found that in pistachio (*Pistacia vera* L.), the K, Zn, and Fe contents of the leaves are affected by rootstock and scion combinations. Akbari scion (*Pistacia vera* L.) budded onto Badami rootstock (*P. vera* L.) efficiently absorbed K and Zn (1.56% and 11.05 ppm, respectively), whereas minimal amounts of K and Zn (0.80% and 7.33 ppm, respectively) were found in Akbari scion budded onto Daneshmandi rootstock (*P. vera* L.). Barg-seyah scion (*P. vera* L.) budded onto Kalle-ghouchi rootstock (*P. vera* L.) absorbed the largest amount of Fe (241 ppm) and Cu (12.15 ppm) among all rootstock and scion combinations.

Ibacache and Sierra (2009) demonstrated that rootstocks significantly affect grapevine nutrition. They studied the effect of 10 rootstocks on grapevines and found significant differences in nutrient concentration in the petioles of four scion cultivars. The petiole P levels doubled in all scion cultivars when Salt Creek rootstock (*Vitis vinifera* L.) was used. Potassium was also increased by up to 5–155% by rootstocks in all scion cultivars. Similarly, N levels increased to 67% in Flame Seedless (*V. vinifera* L.), 77% in Red Globe (*V. vinifera* L.), 33% in Thompson Seedless (*V. vinifera* L.), and 8.5% in Superior Seedless (*V. vinifera* L.) scions compared with self-rooted vines. Bavaresco et al. (2003) utilized two rootstocks for grapevines and observed that the hybrid rootstock 41 B (*V. vinifera* × *V. berlandieri*) significantly increased the nutrient uptake and whole plant dry mass (24%) compared with 3309 C (*V. riparia* × *V. rupestris*). The total N, P, K, Ca, and Mg contents of the plant increased by 38, 21.5, 18, 35, and 12%, respectively. Additionally, the concentrations of micronutrients (Zn, Mn, and B) increased by 16, 94, and 18%, respectively, whereas Cu slightly decreased (1%). Lecourt et al. (2015) observed that rootstocks significantly affect the plant growth (dry matter) and concentration of macro and micronutrients in the leaves and root tissues of grapevine (V. *vinifera* L.). Cabernet Sauvignon (*V. vinifera* L.) grafted onto Riparia Gloire de Montpellier (*V. riparia* L.) increased the leaf N (3%), K (44%), S (70%), Ca (27%), Mg (58%), Fe (65%), Zn (57%), B (93%), Mn (83%), and Al (46%) concentrations compared with Cabernet Sauvignon grafted onto 1103 Paulsen (*V. rupestris* × *V. berlandieri*) irrigated with 2.45 mmol N supply. Habran et al. (2016) investigated the effect of N and rootstock on N uptake and their effect on secondary metabolism in grapes. Cabernet Sauvignon (*V. vinifera* L.) grafted onto 110 Richter increased the leaf and petiole N concentration by up to 14% and 1%, and 9% and 30% under low and optimum levels of N supply, respectively, compared with Cabernet Sauvignon grafted onto Riparia Gloire de Montpellier (*V. riparia*). Rootstocks also affected vine growth (14–16%), berry weight, and composition at the time of harvest. Zamboni et al. (2016) recently declared M1 rootstock [106-8 (*V. riparia* × *V. cordifolia* × *V. rupestris*) × Resseguier n.4 (*V. berlandieri*)] as superior over M3 [R 27 (*V. berlandieri* × *V. riparia*) × Teleki 5C (*V. berlandieri* × *V. riparia*)], 1103P (*V. berlandieri* × *V. rupestris*), and 101–14 (*V. riparia* × *V. rupestris*) rootstocks because of its balanced leaf nutrient profile, nitrogen use efficiency, and improved vine growth (30%). Various rootstocks behave differently in terms of yield, physiological responses, and severity of Fe deficiency; proper rootstock selection can mitigate nutrient deficiency symptoms and responses in grapevines (Covarrubias et al., 2016).

## Vegetable crops

Grafting in vegetable crops is a relatively new procedure that has gained popularity in the last few decades (Lee et al., 2010). Similar to fruit crops, melon (*Cucumis melo* L.) cultivars Yuma and Gallicum grafted onto three *Cucurbita maxima* (Shintoza, RS-841, and Kamel) rootstocks show a significant variation in macronutrients in leaf tissues. Grafting increases the yield (88%–121%) and concentration of P and N in the leaves by up to 6–58% and 6–81%, respectively, compared with self-rooted melon plants (Ruiz et al., 1997). Zhang et al. (2012) found that P uptake and utilization in grafted watermelons are better than those in self-rooted watermelons under low P supply. Various rootstocks perform differently; watermelon grafted onto pumpkin (*Cucurbita moschata* Duch.) performs better than watermelon grafted onto bottle gourd (*Lagenaria siceraria* Standl.). Huang et al. (2013b) found that grafting of watermelon onto Hongdun (*Citrullus lanatus* sp.) and Jingxinzhen No.4 (*C. moschata* Duch.) increases K uptake and whole plant dry mass (1.82 fold) and induces low K tolerance in the watermelon scion. Uygur and Yetisir (2009) worked on watermelons and concluded that the watermelon [*C. lanatus* (Thunb.) Matsum and Nakai] cultivar Crimson Tide grafted onto gourd rootstock (*L. siceraria* Standl.) landraces significantly increases N and K uptake from the soil, especially under salt stress conditions. The concentration of P in the shoots of grafted plants is almost twice that in non-grafted watermelon plants. Therefore, bottle gourds, especially the *Lagenaria* type, can be utilized as a rootstock for watermelon under saline conditions.

Comprehensive mineral nutrient analysis has shown that the concentration of macro and micronutrients in the leaves, fruit rind, fruit flesh, and seeds of grafted watermelon is altered by gourd rootstocks [Ferro and RS841 (*C. maxima* × *C. moschata*) and Argentario and Macis (*Lagenaria* hybrid)]. In leaves, rootstocks increase the N, K, Ca, and Mg concentrations by up to 12–28, 8–36, 13–81, and 12–300%, respectively, and reduce the concentrations of P, Fe, Zn, Mn, and Cu. In fruit rind, flesh, and seeds, rootstocks increase the concentration of all nutrients, thereby proving the superiority of specific graft combinations over self-rooted plants (Yetisir et al., 2013). Watermelon cultivar Zaojia 8424 [*C. lanatus* (Thunb.) Matsum and Nakai] grafted onto bottle gourd cv. Jingxinzhen No.1 (*L. siceraria* Standl.) and pumpkin cv. Qingyanzhen No.1 (*C. maxima* × *C. moschata*) increases the total uptake (mg/plant) and concentration [mg g^−1^ dry weight (DW)] of N, K, Ca, Fe, Mg, and Mn in the roots, stems, leaves, fruit rind, and flesh of grafted plants. The total nutrient uptake of plants grafted onto bottle gourd and pumpkin increases by 30.41 and 49.14%, respectively, at the fruit development stage and by 21.33 and 47.46%, respectively, at the fruit maturation stage compared with non-grafted watermelon plants (Huang et al., 2016a). Another experiment showed that pumpkin rootstock (*C. moschata* Duch.) increases the Mg^2+^ uptake (39%) and whole plant dry mass (1.71 fold) of watermelon plants compared with self-grafted plants under low Mg conditions (Huang et al., 2016b). The *C. maxima* variety Dulce Maravilla clearly increases Fe uptake and translocation to watermelon scions (Rivero et al., 2004).

Grafting is useful in manipulating plant growth and is used in multiple ways. Zhang et al. (2010) reported that when cucumber plants (*Cucumis sativus* L. cv. Xintaimici) are grafted onto *Cucurbita ficifolia*, the uptake of Cu from the soil under Cu stress conditions is significantly reduced. The concentration of Cu in the stem and leaves of the scion is lower in grafted plants than in self-rooted plants; moreover, the concentration of Cu in the roots of grafted plants is higher. This result suggests that the *C. ficifolia* rootstock acts as a Cu accumulator and reduces Cu transport to the scion. However, its sensing, signaling, regulation, and withholding mechanisms still require further explanation. Grafting increases the uptake of K and limits the transport of Na and Cl ions to the scion compared with self-rooted plants under salt stress conditions in cucumber. Grafting also enhances nutrient utilization efficiency as manifested by improved shoot biomass production, yield, and fruit quality (Rouphael et al., 2008; Zhu et al., 2008; Albacete et al., 2009, 2015a,b; Huang et al., 2009; Colla et al., 2012, 2013; Farhadi and Malek, 2015; Gao et al., 2015).

Grafting of tomato (*Solanum lycopersicum* L.) onto Maxifort rootstock (*S. lycopersicum* L. × *Solanum habrochaites*) improves scion growth (11%) and concentration of macro and micronutrients (Table 1). However, different forms of nitrogen (${\text{NO}}_{3}^{-}$, ${\text{NH}}_{4}^{+}$) exert a significant effect on the uptake of other elements; for example, increased availability of ${\text{NH}}_{4}^{+}$reduces the uptake of Ca and Mg in plant tissues (Borgognone et al., 2013). Savvas et al. (2009) reported that grafting tomato onto He-Man rootstock (*S. lycopersicum* × *S. habrochaites*; Syngenta Seeds, Basel, Switzerland) improves fruit yield (11.5%) and increases the concentrations of Ca, K, and Zn by 18, 8, and 11%, respectively, in tomato leaves and reduces the uptake of Mg (27%) and Fe (27%), especially under low levels of Mn. In pepper and eggplant, rootstocks also show a positive response by modifying ion uptake and acquisition of macro and micronutrients and by providing protection from heavy metals (Leonardi and Giuffrida, 2006; Arao et al., 2008; Penella et al., 2015).

**Table 1:** **Effects of rootstocks on the nutrient concentration and growth/yield of grafted plants**.

**Name of crop**	**Name of rootstock**	**Plant part used for determination**	Increase in nutrients concentration (%)^*^	Reduction in nutrients concentration (%)^*^	Yield improvement (%)^*^	**References**
Kinnow *(Citrus reticulata* Blanco.)	Nine different citrus rootstocks were used	Leaves	N (4–18), P (11–77), K (3–43)	–	12.5–563^a^	Ahmed et al., 2007
Newhall navel orange (*Citrus sinensis* Osb.)	Citrange [*C. sinensis* (L.) Osb. × *P. trifoliata* (L.) Raf.] and trifoliate orange [*Poncirus trifoliata* (L.) Raf.]	Mature leaves	B (55)	–	3^b^	Liu et al., 2011
		New leaves	B (51)			
		Roots	B (63)			
Apple (*Pyrus Malus*)	Six different *Malus* rootstocks	Leaves	K (25)	–	60^c^	Cong et al., 2014
	M9, MM106, MM111 Apple, and *Malus domestica* cv. Local	Leaves	N (5–34), Mg (5–68), Zn (10–39), Fe (4–34) Mn (11–70)	K (6–19), Ca (10–28)	44^d^	Amiri et al., 2014
Grapes (*Vitis vinifera* L.)	Different grape rootstocks	Petioles	N (8.5–77), P (5–186), K (5–155)	–	–	Ibacache and Sierra, 2009
	41 B (*V. vinifera × V. berlandieri*) and 3309 C (*V. riparia × V. rupestris*)	Whole plant	N (38), P (21.5), K (18), Ca (35), Mg (12), Zn (16), Mn (94), B (18)	Cu (1)	24^b^	Bavaresco et al., 2003
	Different grape rootstocks	Leaves	N (10–31), P (5–41), K (19–23), Ca (3–20), Mg (26–34), Fe (1–20), B (6–70)	–	30^e^	Zamboni et al., 2016
	Riparia Gloire de Montpellier (RGM) and 110 Richter (*V. vinifera* L.)	Leaves	N (1–14)	–	14–16^e^	Habran et al., 2016
		Petioles	N (9–30)			
	Riparia Gloire de Montpellier (RGM) and 1103 Paulsen (*V. vinifera* L.)	Leaves	N (3), K (44), S (70), Ca (27), Mg (58), Fe (65), Al (46), Mn (83), Cu (43), Zn (57), B (93)	P (33)	152–205^e^	Lecourt et al., 2015
Avocado (*Persea americana*)	Duke and Topa Topa Avocado (*Persea americana*)	Whole plant	N (10), P (5.72), K (2.44), Fe (19), Cu (12.5), Mn (15)	–	5^d^	Slowik et al., 1979
Pistachio (*Pistacia vera* L.)	Badami Pistachio (*Pistacia vera* L.)	Leaves	K (95), Zn (51)	–	–	Sherafati et al., 2011
Melon (*Cucumis melo*)	*C. maxima* × *C. moschata* (Shintoza, RS-841, and Kamel)	Leaves	N (6–81), P (6–58), S (1–4), Mg (1–3), Ca (0–5)	K (4–38)	88–121^d^	Ruiz et al., 1997
	Shintoza (*C. maxima* × *C. moschata*) and Sienne^**^ (Cantaloupe type melon)	Leaves	N (27), P (36), K (28), Ca (39), Mg (16), Mn (35), Zn (31), S (5)	Na (232), Cu (566), B (240), Fe (320)	1–26^a^, 11–24^d^	Bautista et al., 2011
	*Cucurbita maxima* Duchesne × *Cucurbita moschata* (pumpkin, squash)	Leaves	Ca (6), Mg (5)	Na (255), K (9)	11.5^d^	Edelstein et al., 2005
	Shintoza (*C. maxima* × *C. moschata*)	Leaves	N (21), P (17), K (19)	–	255^d^	Salehi et al., 2014
Watermelon (*Citrullus lanatus*)	Hongdun (*C. lanatus* sp.)	Xylem sap	K (246)	–	182^b^	Huang et al., 2013b
	Jingxinzhen No.4 (*Cucurbita moschata* Duch.)	Leaves	Mg (39)	–	171^b^	Huang et al., 2016b
	Ferro, RS841 (*Cucurbita maxima* × *C. moschata*), and Argentario and Macis (*Lagenaria* hybrid)	Leaves	N (12–28), K (8–36), Ca (13–81), Mg (12–300)	–	–	Yetisir et al., 2013
	*Cucubirta maxima* var. Dulce maravilla	Leaves	Fe (193)	–	–	Rivero et al., 2004
		Roots	Fe (143)			
Tomato (*Solanum lycopersicum)*	AR-9704 Tomato (*Solanum lycopersicum* L.)	Leaves	P (68), S (15)	Na (62), Cl (28)	–	Fernández-García et al., 2002
	Maxifort (*S. lycopersicum* L. × *S. habrochaites* S. Knapp and D. M. Spooner)	Leaves	N (0.5), P (13), Ca (10), Fe (9), Mn (3), Cu (18), Zn (17), B (3)	Mg (10)	2^d^, 11^b^	Borgognone et al., 2013
	He-Man (*Solanum lycopersicum* × *Solanum habrochaites*)	Leaves	Ca (18), K (8), Ca (11)	Mg (27), Fe (27)	11.5^d^	Savvas et al., 2009
	*Solanum lycopersicum* cv. Tmknvf2	Leaves	Fe (11.18)	–	–	Rivero et al., 2004
		Roots	Fe (185)			
	*Solanum lycopersicum* Mill. cv. Radja	Leaves	K (13)	–	20–30^d^	Estañ et al., 2005
	Root localized *IPT*-expressing tomato rootstock (35S::IPT) (*Solanum lycopersicum* Mill.)	Leaves	K (20)	Na (30)	30^d^	Ghanem et al., 2011
Rose (*Rosa centifolia*)	Seven *Rosa* rootstocks	Leaves	N (3–12), P (3–23), K (13–31), Ca (7–25), Mg (8–30), Zn (2–18), Mn (141–410), Fe (3–15), Cu (5–36)	–	223^f^	Gammon and McFadden, 1979

* *Compared with self-rooted, self-grafted, or poorly performing rootstock*.

** *Intermediate scion in the process of double grafting*.

a *Total number of fruits per plant*.

b *Whole plant dry mass*.

c *Shoot dry mass*.

d *Fruit yield per plant on weight basis*.

e *Pruning weights of grapevines*.

f *Flower value index*.

Rootstocks play a pivotal role in fertilizer management because they can ensure the justified utilization of available or applied fertilizers in the soil. Harvestable yield is an important criterion of nutrient utilization efficiency. An overview of reported percentage growth and yield increments obtained through grafting among various vegetables is presented in Table 1.

## Ornamental crops

Previous studies on ornamental crops have elucidated the effects of rootstock on the nutrient uptake of such crops. Gammon and McFadden (1979) grafted rose onto seven different rootstocks and determined that rootstock combinations significantly increase nutrient uptake and accumulation. *Rosa fortuniana* Lindl. triggered Mn accumulation five times more than *Rosa odorata;* however, *R. odorata* was superior to *R. fortuniana* in terms of K accumulation. Niu and Rodriguez (2008) evaluated the performance of four rose rootstocks [Dr. Huey (*Rosa hybrida* L.), *R. fortuniana* Lindl., *Rosa multiflora* Thunb., and *R. odorata* (Andr.) Sweet] under chloride-or sulfate-dominated salinity and observed that rootstocks behave differentially in terms of Ca, Mg, K, Na, and Cl uptake. *R. multiflora* accumulates more Na than *R. odorata*, and *R. fortuniana* accumulates the least amount. However, *R. multiflora* retains most of Na in the roots, whereas *R. odorata* transports 57% of the absorbed Na to the shoots. According to another study, commercial rose varieties grafted onto different rootstocks significantly differ in macro (P, K, and Mg) and micro (Cl, Mn, Fe, B, Zn, and Na) nutrient concentrations (Cabrera, 2002). The N use efficiency (NUE) in tobacco can be improved by grafting it onto *Nicotiana tabacum* H-20 rootstock (Ruiz et al., 2006). Several details on increased nutrient uptake through grafting are summarized in Table 1. These pieces of evidence demand the careful selection of the rootstock to enhance nutrient use efficiency in ornamental crops. Grafting is also utilized to produce bonsai plants (an important category of ornamentals); in bougainvillea and several other ornamentals, it is also used to produce plants bearing flowers of different colors or multicolored flowers on the same plant to enhance anesthetics (Relf, 2015). In a recent report, Zhang et al. (2013) observed increased productivity and rooting ability of cuttings obtained from grafted chrysanthemum. In the near future, an increase in the use of grafting is expected in ornamental crops. However, serious efforts are required for the selection of compatible rootstocks in ornamental plants.

## Mechanism

Rootstocks modify the ion uptake in grafted plants. The final concentration of nutrients is a result of uptake, transport, recirculation, and growth. The absorption of nutrients from the soil is affected by numerous factors, including soil properties (pH, cation exchange capacity, and concentration of the nutrients) and root characteristics (root architecture, organic acids and metabolites exudation capacity, and transport ability). This article focuses on root-related mechanisms.

### Root system architecture and ion uptake

In cereal crops, modification of the root architecture is a popular means to enhance ion uptake, accumulation, and utilization efficiency, and this means has been discussed in several reviews (Meister et al., 2014; Rogers and Benfey, 2015; Wissuwa et al., 2016). Nutrient uptake and utilization in horticultural crops are enhanced by selecting appropriate rootstocks. Rootstocks play a vital role in manipulating the nutrient status of the scions by directly affecting ion uptake and transport (Amiri et al., 2014). The ion concentration in the roots and shoots of grafted grapevines depends on rootstock genotype (Lecourt et al., 2015). The selected rootstocks have a vigorous root system, i.e., large main roots, many lateral roots and root hair, large total root length, and root surface area. These roots absorb a large amount of water and nutrients by exploring wide and deep soil volumes (Pérez-Alfocea, 2015). According to a report, the root dry weight of watermelon grafted onto Jingxinzhen No. 4 pumpkin (*C. moschata* Duch.) is 2.24 times that of self-rooted plants (Huang et al., 2013b), and the K uptake efficiency of grafted plants is 2.02 times that of self-rooted plants. Similarly, the root dry weight of a rose cultivar (*Rosa* “BAIore”) is thrice that of a poorly performing cultivar *Rosa* “Frontenac” under stress conditions (Harp et al., 2015). Therefore, this vigorous root system of rootstock can capture and transport a large amount of nutrients to the above ground scion. Vigorous rootstocks have high levels of sugars, amino acids, and enzymes and secrete organic acids in the soil, which are important in nutrient mobilization and affect nutrient availability and uptake (Jaitz et al., 2011; Khorassani et al., 2011; Dam and Bouwmeester, 2016). Jiménez et al. (2011) reported that high concentrations of root sucrose, total organic and amino acids, and phosphoenolpyruvate carboxylase activity in the roots of *Prunus* rootstocks subjected to iron deficiency promote root growth and trigger iron uptake. Similarly, under iron-deficient conditions, the roots of *Malus* species secrete acids, reduce the pH of rhizosphere, and significantly increase the activities of root ferric chelate reductase (FCR), thereby leading to increased ferrous uptake (Zha et al., 2014).

### Transporters and ion uptake

Rootstocks modify ion uptake and transport to the scion by affecting the activities of ion transporters. Transporters are involved in ion uptake, root compartmentation, and translocation of these absorbed ions to aboveground plant parts. Transporters are highly specific under different environmental conditions and in the concentrations of specific nutrients present in the soil. For example, in two citrus rootstocks [Carrizo citrange (*C. sinensis* [L.] Osb. × *P. trifoliata* [L.] Raf.) and Cleopatra mandarin (*C. reshni* Hort. ex Tanaka)], eight different K transporters have been identified (Caballero et al., 2013). Grafting affects the activity of ion transporters. The expression levels of Mg transporter genes *MGT1, MGT3, MGT4, MGT5*, and *MGT7* are much higher in the roots of Jingxinzhen No.4 pumpkin (*C. moschata* Duch.) used as rootstock compared with the roots of self-rooted watermelon cultivar Zaojia 8424 [*C. lanatus* (Thunb.) Matsum and Nakai], especially under low Mg conditions. These higher expression levels enhance the Mg^2+^ uptake of watermelon grafted onto Jingxinzhen No.4 pumpkin rootstock (Huang et al., 2016b). However, the signaling mechanism in the activation of these genes still requires further investigation. Similarly, the activities of ferric-related uptake and transport genes (*NAS1, FRD3*, and *NRMAP3*) are significantly increased under iron-deficient conditions in apple rootstock *Malus xiaojinensis*, thereby increasing the ferrous uptake (Zha et al., 2014). Han et al. (2009) reported that a genetically engineered bottle gourd (*L. siceraria* Standl.) rootstock having a modified Arabidopsis Ca^2+^/H^+^ exchanger *sCAX2B* improves the biomass and quality of watermelon fruits by enhancing nutrient transport to the scion. This transporter provides enhanced Ca^2+^ transporter substrate specificity and decreases Mn transport capability. Gonzalo et al. (2011) studied the response mechanism of two *Prunus* rootstocks [Myrobalan plum (P 2175) and peach–almond hybrid (Felinem)]. Felinem showed an activated expression of FCR and the iron transporter gene. The local signal induced a quick response of the transporter, whereas the FCR gene was expressed later. By contrast, in P 2175, the response appeared later, and long-distance signals appeared to be involved. However, in both situations, signaling molecules were not identified; this mechanism thus requires further investigation.

Grape rootstocks [SO4 and K5BB (VITIS Rauscedo, Società Cooperativa Agricola, Rauscedo, Italy) favor nitrate uptake by affecting the activities of low-affinity nitrate transporter (*VvNRT1.3like*) and high-affinity nitrate transporter (*VvNRT2.4like*) genes in grapevines (Tomasi et al., 2015). In pear rootstock (*Pyrus betulaefolia*), six ${\text{NH}}_{4}^{+}$ transporter genes (*LjAMT;1, LjAMT;2, LjAMT;3, LeAMT;3, PbAMT1;3*, and *PbAMT1;5*) have been reported (Mota et al., 2008; Li et al., 2016d); however, the roles of these and other transporters in grafted plants remain unclear. In two citrus rootstocks [Cleopatra mandarin (*C. reshni* hort. ex Tanaka) and Troyer citrange (*C sinensis* × *P trifoliata*)], low-and high-affinity transport systems work depending on the availability of nitrate ions in the external medium (Cerezo et al., 2007). However, the sensing and signaling mechanisms (how plants sense the internal and external concentrations of nitrate and respond accordingly) have not been explained in grafted plants. Non-grafted plants utilize a dual-affinity nitrate transporter (CHL1) and protein kinases (CIPK28 and CIPK8) to sense a wide range of nitrate concentration changes in the soil and to alter their own transport properties (Ho et al., 2009). Another recent study revealed that small peptides are produced in nitrogen-starved roots and then transported to the shoot; this root-to-shoot signaling helps plants adopt under prevailing conditions (Tabata et al., 2014). These pieces of evidence are missing in grafted plants and need further study.

### Hormones, miRNAs, and ion uptake

Hormones and miRNAs are the key players that affect nutrient absorption and transport by affecting the root growth and activity of ion transporters. Grafting onto rootstocks changes hormonal levels (cytokinin and auxin) in roots and shoots, triggers growth, delays leaf senescence, and helps tolerate environmental stress (Albacete et al., 2009; Van-Hooijdonk et al., 2010; Ghanem et al., 2011). In a previous study, grafting a transient root IPT induction (HSP70::IPT) and wild type (WT) tomato (*S. lycopersicum* L. cv. UC82-B) scions onto root localized *IPT*-expressing tomato rootstock (*35S::IPT*) grown under salt stress conditions (75, 100 mM NaCl) increased root, xylem sap, and leaf bioactive cytokinin by 2–3 times, increased leaf K concentration (20%), and reduced Na concentration (30%) in the transient root IPT induction scion compared with plants grafted onto non-transformed plants. Similarly, *IPT*-expressing tomato rootstock (*35S::IPT*) increased fruit *trans*-zeatin concentrations 1.5–2 fold and fruit yield (30%) in WT tomato scion compared with WT tomato plants grafted onto non-transformed rootstock (Ghanem et al., 2011). Roots of rootstock-grafted apple with more branching (MB) mutant scion show elevated cytokinin and auxin contents and reduced expressions of *MrPIN1, MrARF, MrAHP*, most *MrCRE1* genes, and cell growth-related genes *MrGH3, MrSAUR*, and *MrTCH4*. Auxin accumulation and transcription of *MrPIN3, MrALF1*, and *MrALF4* induce lateral root formation in MB-grafted rootstock, and these new roots contribute to nutrient absorption (Li et al., 2016a). miRNAs regulate the expression of transporters involved in nutrient uptake and mobilization (Fujii et al., 2005; Chiou et al., 2006; Sunkar et al., 2007; Valdés-Lopez et al., 2010; Paul et al., 2015). A recent report (Li et al., 2016c) showed that under N or P deficiency, the expression levels of 19 miRNA target genes in the roots of pumpkin (*C. moschata* Duch.)-grafted cucumber (*C. sativus* L.) are higher than those in self-grafted cucumber. Many studies have revealed the role of miRNA 399 in P absorption, transport, metabolism, and homeostasis in plants. P deprivation is rapidly transmitted to the shoots where miRNA 399 is produced, conjugates, changes the activity of PHO2 (an essential transporter of phosphate mobilization), and regulates phosphate homeostasis (Pant et al., 2008). The expressions of miRNA 399 and csa-miR-n08 are higher in pumpkin (*Cucurbita moschata* Duch.)-grafted plants. These affect the expressions of *PHR1* and *E3* (ubiquitin–protein ligase), which are essential for P absorption and metabolism. The PHR1 transcription factor binds to the promoter region of many phosphorous-deficient responsive genes, including those encoding phosphate transporters and protein kinases (Rubio et al., 2001; Todd et al., 2004). Under phosphorous starvation, the activity of miR2111 doubles within 3 h, whereas under N deficiency, the opposite occurs (Pant et al., 2008; Xu et al., 2013). Therefore, the relationship among the expression levels of different miRNAs still requires explanation. Several recently published articles have summarized the role of miRNAs (Liu and Vance, 2010; Zeng et al., 2014; Kulcheski et al., 2015; Paul et al., 2015) and ion transporters (Conte and Walker, 2011; Finazzi et al., 2015; Pinto and Ferreira, 2015; Gu et al., 2016) in nutrient acquisition, transport, and homeostasis. However, information on grafted plants is limited and requires the attention of plant biologists because grafted plants are complex in nature, and their responses are influenced by the genetic makeup of the scion and the rootstock and their interaction.

In conclusion, rootstocks modify nutrient availability to the scion. A vigorous root system, increased secretion of root exudates (organic acids and primary and secondary metabolites), enhanced expression of transport-related genes, increased absorption and transport, improved ion homeostasis, and remobilization capacity of the rootstocks ensure improved nutrient supply to the scion. These nutrients enable scions to increase light energy transformation, CO_2_ conductivity, dark reaction activity, and rate of photosynthesis, thereby improving nutrient utilization (Weng, 2000; Sun et al., 2002; Qi et al., 2006; Wei et al., 2006; Huang et al., 2011).

## Rootstock limits the uptake and transport of salts and heavy metals

Rootstocks improve the acquisition of essential elements and reduce the uptake and transport of salts (e.g., Na and Cl) and heavy metals (e.g., Cr, Ni, Cd, Sr, and Ti) through ion exclusion or retention. Reciprocal grafting experiments on cucumber (*C. sativus* L.), pumpkin (*C. moschata* Duch.), and melon (*C. melo* L.) rootstocks revealed that pumpkin is salt tolerant. Pumpkin excludes 74% of the available Na, whereas no Na is excluded by melon. Similarly, Na retention in pumpkin rootstock reduces the level of Na in leaves by 46.9%, whereas no decrease is observed in melon rootstock (Edelstein et al., 2011). Estañ et al. (2005) found that grafting a commercial tomato cultivar Jaguar (*S. lycopersicum* L.) onto tomato rootstocks (Radja, Pera, and the hybrid Volgogradskij × Pera) increases fruit yield by up to 80% by regulating saline ions (Na and Cl). Pumpkin-grafted cucumber shows an increased expression of plasma membrane H^+^-ATPases (*PMA*) and plasma membrane Na^+^/H^+^ antiporter (*SOS1*) under NaCl stress conditions compared with self-grafted cucumber. The increased activities of *PMA* and *SOS1* enable the root to pump Na^+^ from the cytosol of root cells to the external medium (Lei et al., 2014). Citrus rootstock responds differently to Cl stress, and a number of uncharacterized membrane transporter genes, such as *NRT1-2*, are differentially expressed in poor Cl excluder Carrizo citrange (*C. sinensis* × *P. trifoliata*) and efficient Cl excluder Cleopatra mandarin (*C. reshni* hort. ex Tanaka) rootstocks (Brumós et al., 2009). The strong repression of the *ICln*gene in Cleopatra mandarin regulates Cl uptake and transport to the shoot. It reduces the net Cl loading to the root xylem and thus enhances the Cl tolerance of Cleopatra mandarin (*C. reshni* hort. ex Tanaka) (Brumós et al., 2010). Transporters also help enhance salt stress tolerance. Overexpression of a *Malus* Na^+^/H^+^ anti-porter gene improves the salt tolerance of M26 dwarfed apple rootstock (Li et al., 2013). Rootstocks reduce Na and Cl loading and transport to the scion while increasing the uptake of K, Ca, and Mg ions and allow small osmotic potentials with a low energy cost. Therefore, rootstocks increase the tolerance of scion species to salt and toxic elements.

Grafting cucumber (*C. sativus* L.) onto *C. maxima* × *C. moschata* rootstocks reduces the absorption and transport of Cd and Ni to the scion. The absorption of Cd depends on rootstock genotype (Savvas et al., 2013). Heavy metals also affect the availability of essential nutrients; however, the negative effect can be minimized by grafting onto appropriate rootstocks. Root structure (genotype) and transporter activities appear to be involved in the reduction of hazardous ion absorption and transport. In a reciprocal grafting experiment, Xin et al. (2013) found that spinach shoot Cd concentration is unrelated to root Cd absorption; it depends on Cd transport from the root to the shoot. The thick phellem (outer zone of periderm) and outer cortex cell walls of the water spinach (*Ipomoea aquatic* Forsk.) rootstock QLQ retain more Cd in the root compared with water spinach rootstock T308. Apple rootstock (*Malus Baccata* Borkh.) reduces the influx of Cd and shows the least amount of Cd in leaf petioles compared with other apple rootstocks (*Malus hupehensis* Rehd., *M. micromalus* “qingzhoulinqin,” and *M. robusta* Rehd.). *M. Baccata* has a low transcript level for Cd uptake and transport genes (*HA7, FRO2-like, NRAMP1*, and *NRAMP3*) and a strong transcript level for detoxification genes (*NAS1 and MT2*). The reduced expression of Cd transport genes results in reduced uptake and transport of Cd (Zhou et al., 2016). Repression of Cd uptake and transport genes occurs through a local signal or as a result of long-distance signaling, although this issue remains unclear. Thus, investigations about the sensing and signaling mechanisms of heavy metals in grafted plants are required.

## Future perspectives

Although grafting is a centuries-old phenomenon currently practiced at the commercial scale, the physiological and molecular bases of grafting remain unclear. With *Arabidopsis* as a model plant, the basic scientific mechanism of grafting is being investigated. Rootstocks and scions affect each other; their interaction mechanism also requires further attention. Grafting compatibility between the rootstock and scion is important for uninterrupted flow of water, nutrients, and carbohydrates. Grafting incompatibility occurs when either of the plant parts (rootstock or scion) completely or partially fails to grow after successful grafting and in several latter stages of plant growth. Overgrowth of the scion over the rootstock, overgrowth of the rootstock over the scion, and bud union crease are common symptoms of incompatibility. Double grafting can resolve compatibility issues, but it complicates the process of grafted seedling production and increases the production cost. Grafting incompatibility may be related to defense responses. Cookson et al. (2014) observed a coordinated upregulation of stress response-related genes in hetero-grafts compared with self-grafted grapevines (*Vitis* spp.). In a recent study, Melnyk et al. (2015) revealed that auxin response genes *IAA18, IAA28*, and *ALF4* are responsible for vascular reconnection at the graft junction. Although authors have not linked these genes with incompatibility, the roles of these genes in incompatibility cannot be overlooked and require further investigation. Crease formation affects water and nutrient transport; however, to what extent this effect impairs the availability of nutrients to the scion remains unclear. Similarly, different methods of grafting are utilized for fruits and vegetables, but their effects on the quality of the vascular connection, crease development, and nutrient supply have not been considered to date. Detailed histochemical investigations are required to study the cell and vascular tissue structures at graft junctions and their relationship with water and nutrient transport. The rootstock and scion are important, but the rootstock is considered critical. No failure caused by scion variety has been reported in the fruit industry in any country, but a number of examples show failure caused by inappropriate rootstock (Castle, 2010). Therefore, in perennial fruit plants, the choice of proper/compatible rootstock is highly significant because once an orchard is established; it remains productive for a long time. Similarly, selecting compatible and appropriate rootstocks for important vegetables and ornamental crops need consideration. Although large numbers of rootstocks are available and used for different crops, improved rootstocks with improved characteristics, such as multi-disease resistance and enhanced nutrient absorption and utilization efficiencies, are still lacking.

Rootstocks enable increased water and nutrient absorption through efficient uptake systems and/or their capacity to explore wide and deep soil volumes. However, these properties should be transferred to increase yield or improve efficiency. This transfer requires well-designed studies on yield responses vs. a gradient of nutrient/water application to the plant. The accumulation of a given nutrient in the leaves during vegetative growth is not an indicator of nutrient use efficiency and should be considered in relation to the total nutrients absorbed by the plant and the harvestable yield produced (Pérez-Alfocea, 2015). Moreover, the response of rootstocks under different growing conditions changes considerably (Cong et al., 2014). Rootstocks are also inherently selective in the absorption of different nutrients. Fernández-García et al. (2002) observed an increase in the uptake of P and Ca and a decrease in the uptake of N, K, S, and Mg by grafting Fanny and Goldmar (*S. lycopersicum* L.) onto AR-9704 (*S. lycopersicum* L.). Nutrient-specific rootstock breeding is currently eliciting the attention of scientists (Venema et al., 2011). Macronutrients, such as P and K, are finite in nature and costly, so they are of particular interest. With the passage of time, linkage map locations for quantitative trait loci (QTLs) have become available for nutrient uptake and transport. For example, several QTLs controlling the concentration of nutrients in the leaves of tomato and apple rootstocks have been found (Fazio et al., 2013; Asins et al., 2015). These QTLs allow scientists to alter the root architecture of plants as needed. Several transgenic rootstocks have been reported to control fan leaf virus in grapes (Gambino et al., 2010), fungal diseases in citrus (Mitani et al., 2006), pathogen damage in tomato (Haroldsen et al., 2012), and nutrient absorption in tomato and watermelon (Han et al., 2009; Ghanem et al., 2011). Thus, nutrient-efficient transgenic rootstocks are expected to become commercially available for different horticultural crops in the near future. The use of genetically engineered nutrient-efficient rootstocks (transgrafting) and scions will extend the utility of grafting by combining an ancient technique with molecular strategies of the modern era (Goldschmidt, 2014), leading to improved nutrient use efficiency. However, the acceptability of genetically modified crops by the public is a question that needs to be addressed.

Remarkable achievements have been made in the field of ion transporter in *Arabidopsis*, rice, wheat, and other crops. However, a limited number of studies related to the uptake and transport of essential nutrients have been performed on grafted plants. Thus, further study is needed to thoroughly understand the sensing, signaling, uptake, and transport mechanisms in grafted plants. This information will be helpful for breeding programs to develop nutrient-efficient rootstocks. Grafting in horticultural crops can also help understand the basic biology of the grafting mechanism, the reasons for incompatibility, sensing and signaling of nutrients, ion uptake and transport, the mechanism involved in the accumulation of heavy metals in the rootstock, and the restriction of heavy metal transport to the scions. Moreover, vegetable grafting studies can be performed quickly, and the results can be compared with those on self-rooted or self-grafted plants. Imperative conclusions can be obtained for commercial adoption.

Several approaches, such as the use of plant-growth-promoting rhizobacteria (PGPR), can be combined with grafting to further enhance nutrient uptake and utilization efficiency. These PGPR act in diverse ways, including the production or degradation of important plant growth hormones, which in turn controls root growth and affects nutrient absorption (Dodd et al., 2010; Wang et al., 2016b). The use of arbuscular mycorrhizal fungi enhances the acquisition of P, N, Mg, and Ca, maintains the K:Na ratio, affects nodulation and nitrogen fixation, and alters gene expression (*PIP*, Na/H antiporters, *Lsnced, Lslea*, and *LsP5CS*), thereby improving plant growth (Parniske, 2008; Evelin et al., 2009; Kumar et al., 2015; Miceli et al., 2016; Wang et al., 2016b). Single- and double-root grafting of cucumber increase the population of soil actinomycetes (bacteria) and reduce the population of fungi (*Fusarium oxysporum*) (Xie et al., 2012). However, for other crops, whether the rootstock and scion affect each other's soil microbiota and their effect on the nutrient absorption of rootstocks remain largely unknown and thus need further study. The use of natural (humic acid, root exudates, phytosiderophores) and synthetic chelators [ethylenediaminetetraacetic acid (EDTA), [(ethylenediamine-N, N'-bis (2-hydroxyphenylacetic acid) (EDDHA)], diethylenetriamine pentaacetic acid (DTPA)] is reported to enhance nutrient (Fe, Zn, Cu, Mn, etc.) mobilization and phyto-availability; however, the suitability and efficacy of these chelators in grafted plants need investigation (Treeby et al., 1989; Bocanegra et al., 2006). Exogenous application of plant growth regulators (e.g., auxin) and several other novel substances (e.g., melatonin) is currently being considered by scientists to alter root architecture and enhance nutrient uptake (Nawaz et al., 2016). However, further studies are required to standardize the concentration and method of application of these substances for grafted plants.

## Author contributions

MN, MI, QK, and FC wrote the manuscript, YH, WA, and ZB revised, and finally approved the manuscript for submission. All authors declare no competing financial interests.

### Conflict of interest statement

The authors declare that the research was conducted in the absence of any commercial or financial relationships that could be construed as a potential conflict of interest.
